# Travel Patterns in China

**DOI:** 10.1371/journal.pone.0016364

**Published:** 2011-02-02

**Authors:** Tini Garske, Hongjie Yu, Zhibin Peng, Min Ye, Hang Zhou, Xiaowen Cheng, Jiabing Wu, Neil Ferguson

**Affiliations:** 1 MRC Centre for Outbreak Analysis and Modelling, Imperial College London, London, United Kingdom; 2 Office for Disease Control and Emergency Response, Chinese Center for Disease Control and Prevention, Beijing, People's Republic of China; 3 Department of Communicable Disease Prevention and Control, Shenzhen Center for Disease Control and Prevention, Shenzhen, People's Republic of China; 4 Department of Communicable Disease Prevention and Control, Anhui Provincial Center for Disease Control and Prevention, Hefei, People's Republic of China; Stanford University, United States of America

## Abstract

The spread of infectious disease epidemics is mediated by human travel. Yet human mobility patterns vary substantially between countries and regions. Quantifying the frequency of travel and length of journeys in well-defined population is therefore critical for predicting the likely speed and pattern of spread of emerging infectious diseases, such as a new influenza pandemic. Here we present the results of a large population survey undertaken in 2007 in two areas of China: Shenzhen city in Guangdong province, and Huangshan city in Anhui province. In each area, 10,000 randomly selected individuals were interviewed, and data on regular and occasional journeys collected. Travel behaviour was examined as a function of age, sex, economic status and home location. Women and children were generally found to travel shorter distances than men. Travel patterns in the economically developed Shenzhen region are shown to resemble those in developed and economically advanced middle income countries with a significant fraction of the population commuting over distances in excess of 50 km. Conversely, in the less developed rural region of Anhui, travel was much more local, with very few journeys over 30 km. Travel patterns in both populations were well-fitted by a gravity model with a lognormal kernel function. The results provide the first quantitative information on human travel patterns in modern China, and suggest that a pandemic emerging in a less developed area of rural China might spread geographically sufficiently slowly for containment to be feasible, while spatial spread in the more economically developed areas might be expected to be much more rapid, making containment more difficult.

## Introduction

Worldwide urbanisation and increased human mobility create conditions favourable to the spread of emerging pathogens, such as the SARS epidemic [1], [2] and now the influenza H1N1pdm pandemic [3], which spread around the world with unprecedented speed. Understanding the spatiotemporal spread of emerging infections requires detailed information on the epidemiological coupling of human populations. Such coupling is mediated by travel. Hence recent years have seen human travel data incorporated into models of respiratory disease spread to explain the observed patterns of spatial spread [4], and to inform contingency planning [5], [6], [7].

Increasing volumes of travel data for developed countries is becoming available, including origin-destination matrices for commuting obtained from census data, data from surveys and data from mobile phones [8], but much less is known for other parts of the world. Here we analyse a unique dataset, collected in two very different areas of China that gives a snapshot of movement patterns in this rapidly developing country in the early 21^st^ century.

### Study areas

**Figure 1:**
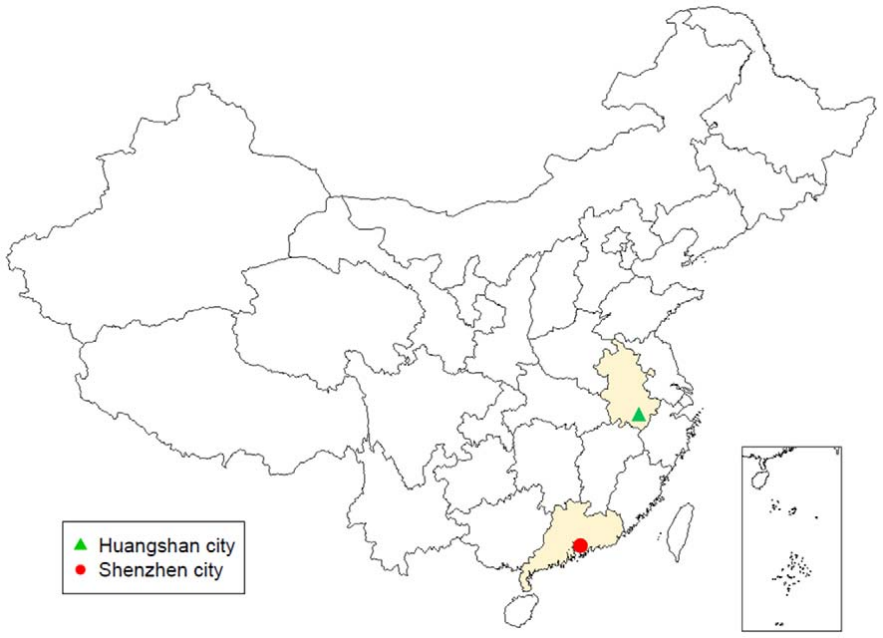
Location of Huangshan city in Anhui province and Shenzhen city in Guangdong province within China.

A survey of commuting and travelling behaviour of 20,000 people from two different regions in China was conducted. Half of the study participants lived in Huangshan city at the southern end of Anhui province, whereas the other half come from in Shenzhen city in Guangdong province, bordering Hong Kong Special Administrative Region (SAR), China (see Figure 1).

Anhui is a mainly agricultural and relatively poor province. Huangshan city has a population of 1,470,000, comprising both urban and rural areas. The population density overall is 180 people/km^2^.

By contrast, Guangdong province has a rapidly growing population and is now the largest province by population; it is also the richest province. Shenzhen city is a Special Economic Region, meaning it is more open to trade and commerce than other parts of China. Since it was established in the early 1980s, it has grown to a population officially recorded as 8.6 million at the end of 2007 with an average population density of 4200 people/km^2^. Shenzhen is a major manufacturing centre in China, but industries also include finance and high-tech enterprises. The population can be divided into local residents who tend to be better educated, and lower-skilled migrant workers. The latter typically originate from other provinces and come into Shenzhen for at least 6 months of the year, living in dormitories provided by their companies.

## Methods

### Ethics statement

The Institutional Review Board of China CDC granted ethical permission for the study. Oral informed consent was obtained from all adult participants and parental consent for minors at the time of interview.

### Study design

10,000 individuals were selected each from the administrative areas of Huangshan city in Anhui province and Shenzhen city in Guangdong province.

Huangshan city consists of 3 urban districts and 4 rural counties. Study participants were selected from one urban district, Tunxi district, and one rural county, Xiuning county, in proportion reflecting the overall proportions or urban and rural citizens within Huangshan city. Within Tunxi district, households were sampled randomly from all 11 communities, whereas within Xiuning county, households were sampled randomly from 30 of the 259 villages in the county.

Shenzhen city consists of 6 urban districts with a total of 639 communities. A substantial proportion of the population are migrant workers who live in company provided dormitories. From 30 communities within Shenzhen city, households and migrant workers were recruited for the study in numbers reflecting the proportion of migrant workers within the population.

All members of the selected households were administered a questionnaire, provided in Text S1, including demographic information (age, sex, family size, occupational status, local registration status) as well as distance typically travelled to school or work. In addition, participants were asked to enumerate any non-commuting journeys undertaken in the last week to destinations outside the study area.

### Analysis

We analysed the demographic data, commuting distances and travel behaviour. In order to find predictors of the commuting distances, we fitted linear regression models and characterised the remaining variability within the categories defined by the best fitting regression model by fitting functional distributional forms to the observed distributions of distances travelled to school or work. To model the probability of an individual travelling we fitted logistic regression models to the data. In order to describe the distances of the occasional journeys we fitted functional forms to the overall distributions of distances travelled for the different cities using a gravity model approach. Due to the large number of possible origins and destinations within the country, we used a gravity model integrated over individual destinations that only retained information on the distances travelled.

#### Predictors of the distance travelled to school or workplace

In order to find predictors of the distance travelled to school or workplace we fitted linear regression models to the data, adjusting the confidence intervals of the estimated parameters for the clustering by households in the dataset. We used the logarithm of the distance to the school or workplace as the independent variable. For those individuals with a reported distance of 0 km we used ln(uniform[0,0.5[)) instead. Potential independent variables are age, sex, family size, registration status, frequency of travel outside the study area and whether or not they live in a rural area (Huangshan) or are migrant workers (Shenzhen). For students, age was categorised by type of school, whereas for employees we used 10-year age bands, with those below 20 and those over 60 merged. Family size was classified into small (< = 3) and large (>3) families. To decide which variables and interactions to include into the final model, we fitted models with all possible variable combinations, and up to two interaction terms, as well as all possible combinations of interaction terms for all combinations of up to 4 variables. The model with the lowest value of the Bayesian information criterion 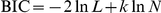 [9], where k is the number of parameters in the model and N is the number of observations, was selected as final model. The data were then further stratified by the variables identified in the linear regression, and the remaining variability of commuting distances within each stratum was characterised by fitting a functional form to the distance distributions separately for each stratum with at least 5 individuals.

#### Characterising population heterogeneity in commuting behaviour

We wished to characterise the variation between individuals in the typical distances travelled to school or work. To this end we categorised observed log distances into bins of uniform width, with the first category containing all distances up to 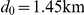, and subsequent distance cut-offs increasing by a factor of 1.5, such that 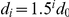. Lognormal distributions with a complementary cumulative distribution function 
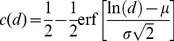
(1)were fitted to the observed distance distributions using maximum likelihood estimation. Here, *µ* and *σ* are the mean and standard deviation of the log distance of the fitted distribution.

We also included an additional parameter 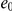 determining the excess of distances observed within the lowest distance category, such that the probability of observing a distance in category 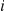 is given by 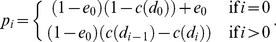
(2)


#### Predictors of patterns of occasional travel

To find predictors of those who had travelled outside the study area within the past week, we fitted logistic regression models to the data analogously to the way described above for the school and workplace distances. Here we did not perform a separate analysis for students and employees, so the occupational status was included as an additional potential independent variable. We fitted models with all possible variable combinations and up to one interaction term, and all possible combinations of interaction terms, if up to 3 variables were included.

#### Analysis of the distance distributions of occasional travel

For the occasional journeys we fitted a simplified gravity model, where we assumed that the number of journeys from each study area to destinations a certain distance 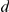 away is determined by the population density 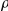 at that destination as well as the distance itself.

Gravity models assume that the frequency of journeys 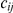
from an origin 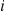 to a destination 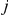 with population sizes 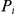 and 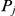 that are a distance 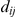 apart can be described by 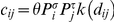. Here, we had data on only two different origins, and the number of observed journeys was small compared to the possible number of destinations within Mainland China, Hong Kong and Macao, preventing the robust analysis of a full gravity model that would have described the spatial anisotropy given by the population density. We therefore binned the distances into bins of logarithmic width and summed over all destinations within each distance category. As the origin was not known precisely, but only as a point somewhere within each study area, we also integrated over the area of origin, weighted by the local population density. We restricted the model to values of 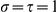 of the population powers on populations at both origin and destination as this yields an easy interpretation within an individual based framework (the case of 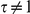 is investigated in Text S2), such that the probability of a journey having a distance 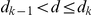 is given by
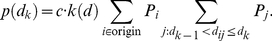
(3)


As spatial kernel 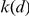
we used again a lognormal distribution as given in equation (1), albeit without the additional parameter for excess of short journeys. When fitting gravity models to mobility data, parameter estimates frequently depend on the aggregation level of the data used [10]. We therefore performed sensitivity analyses for the bin widths used in the aggregation, which are given in Text S2. The distance distributions were extracted from the recorded journey destinations as described in Text S2.

95% confidence intervals for the parameter estimates of the fitted distributions to both the commuting and occasional journey distances were obtained by varying all parameters around the maximum likelihood estimate and identifying the part of the parameter space, where twice the negative log likelihood differs less than 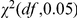, the chi-squared value on the relevant degrees of freedom corresponding to 95% confidence, from the optimum likelihood, as described in [11].

## Results

### Characteristics of the study populations

The selected study participants in Huangshan were interviewed between 21^st^ July and 8^th^ September 2007, in Shenzhen between 9^th^ and 30^th^ August 2007. Some descriptive statistics of the study populations are shown in Table 1. For a comparison of some demographics between the study populations and the overall Chinese population, see Text S2. It is notable that the Shenzhen migrant workers have a high proportion (58%) of women. They live in dormitories, but no data was collected on the number of people sharing a dormitory. In the other populations, family sizes vary between 1 and 12, the largest mean family size is found in rural Huangshan (3.68), and slightly smaller mean family sizes in urban Huangshan (3.25) and Shenzhen locals (3.08). The proportion of the population with residency permit differs markedly between cities; it is high in Huangshan (84% urban, 99.2% rural), but low in Shenzhen (21% locals, 0.95% migrants).

**Table 1:** Descriptive statistics of the study populations, showing the number of individuals (N) and percentage of the population (exact binomial 95% confidence interval), or mean (range).

	Huangshan urban		Huangshan rural		Shenzhen local residents		Shenzhen migrant workers	
	N	% (95% CI)	N	% (95% CI)	N	% (95% CI)	N	% (95% CI)
Total	2317		8126		9894		1994	
**Sex**								
male	1115	48 (46–50)	4059	50 (49–51)	5173	52 (51–53)	834	42 (40–44)
**occupation**								
student	439	19 (17 -21)	1239	15 (14–16)	1332	13.5 (12.8–14.2)	0	0 (0–0.002)
employee	1160	50 (48–52)	5832	72 (71–73)	5955	60 (59–61)	1994	100 (99.8–100)
unemployed	718	31 (29–33)	1055	13 (12–14)	2607	26 (25–27)	0	0 (0–0.002)
**registration**								
registered	1957	84 (83–86)	8062	99.2 (99.0–99.4)	2074	21 (20–22)	19	0.95 (0.57–1.5)
**family size**								
mean family size (range)		3.25 (1–8)		3.68 (1–11)		3.08 (1–12)		n/a
**travelling**								
travelled in past week	224	9.7 (8.5–11)	233	2.9 (2.5–3.3)	360	3.6 (3.3–4.0)	30	1.5 (1.0–2.1)
mean no of journeys if travelled (range)		1.28 (1–7)		1.09 (1–7)		1.06 (1–5)		1.03 (1–2)
**mean age (range)**		35.9 (0–98)		40.9 (0–94)		30.7 (0–94)		25.4 (15–70)

**Figure 2:**
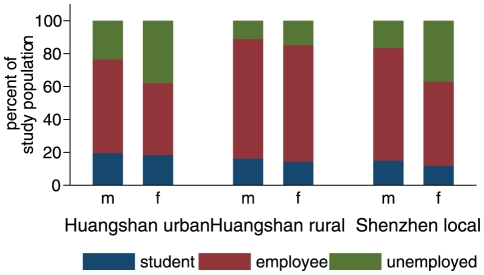
Percentage of students, employees and unemployed or retired by gender for the different study populations.

**Figure 3:**
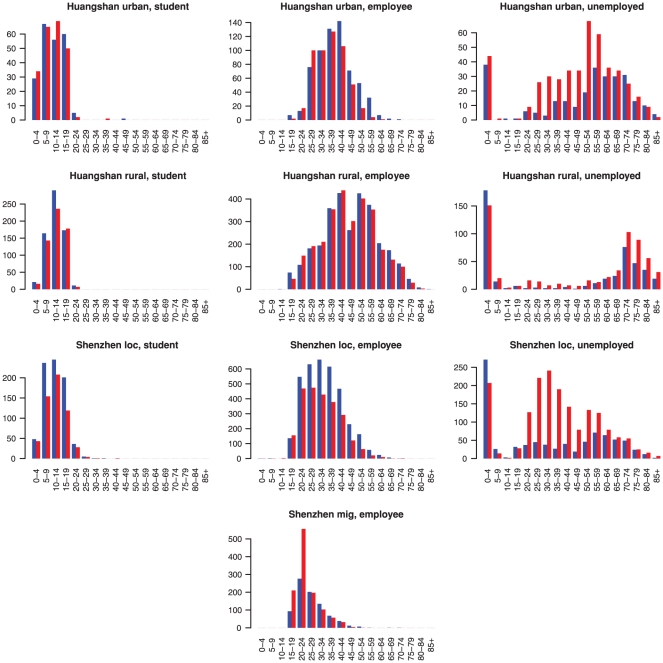
Age distributions for the different occupational groups and study populations by gender. Blue = males, red = females.

There are interesting differences in occupational status by sex and age, see Figure 2 and Figure 3. The proportion of students is similar across the Huangshan and Shenzhen local residents populations, with little differences between males and females. However, the proportion of the unemployed or retired is low for both genders in rural Huangshan, whereas in urban Huangshan and the Shenzhen local population, unemployment is higher, with a pronounced excess in women. From the age distributions in Figure 3 one can see that those not working are made up predominantly of young children and the elderly, and, in urban Huangshan and Shenzhen, of women in the 20–60 year age range, who are probably running the household. Overall the Shenzhen population is very young, particularly the migrant workers.

### Commuting distances

**Figure 4:**
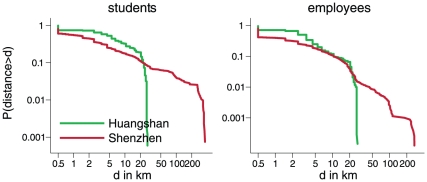
Cumulative distributions of the distances to schools and workplaces for the different study populations.

The distance distributions for both students and employees from Huangshan city fall off very steeply between 20 and 30 km, whereas the distance distributions from Shenzhen city show a longer tail of up to 300 km (Figure 4). We examined the covariates which predicted distance travelled to work or school using linear regression (see Methods). In Huangshan, the best fit model for students included age and rural/urban area; the best model for employees included age, rural/urban area and sex. Fitting the rural and urban areas separately yielded best fit models retaining the same variables for both types of area.

**Figure 5:**
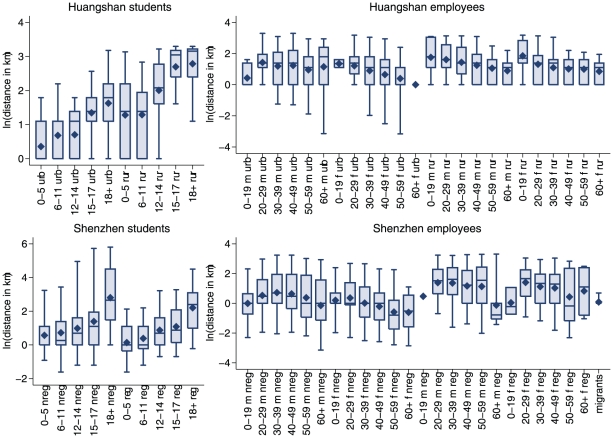
Commuting distances stratified by the variables identified in the regression analysis. The data is displayed separately for students and employees in the different study areas as box plots with mean (diamonds), median (horizontal lines), interquartile range (bars) and 90% spread (whiskers). Categories are labelled by age, gender (m = male, f = female), urban (urb) or rural (rur) area, and registration status (reg = registered, nreg = not registered).

For Shenzhen, the best model for students included age and registration status; the best model for employees included age, sex, registration status and whether an individual was a local resident or migrant worker. Fitting the local population and migrant workers separately yielded the same included variables for the local population, but the preferred model for the migrant population was the null model, indicating a fairly homogeneous composition of this subpopulation. The R^2^-values of all these fits were low to moderate for students but very low for employees (R^2^ = 0.40, 0.14, 0.037, 0.061 for Huangshan and Shenzhen students, and Huangshan and Shenzhen local employees, respectively), indicating substantial residual variation in distances within categories. The observed distance distributions by category are summarised in Figure 5.

Overall, age is the most important predictor for students with older students travelling further to school. In Huangshan, students from rural areas have longer distances than those from urban areas, whereas in Shenzhen, those not registered travel longer distances than those registered.

For employees, men tend to have a longer commuting distance than women. Here, the commuting distance tends to decrease with age, but teenage employees in the Shenzhen local resident population travel fairly short distances. In contrast to the situation for students, local registration is associated with longer commuting distances in the Shenzhen local employees. Further details about the regression models can be found in Text S2.

**Figure 6:**
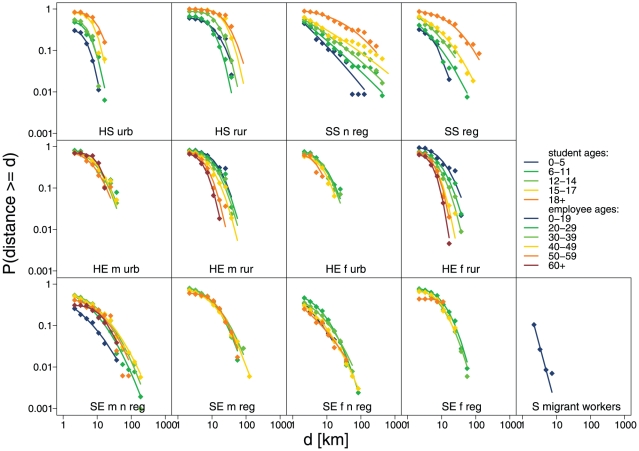
Observed and fitted cumulative distance distributions stratified by the variables identified in the regression analysis. Diamonds  =  observed distributions, lines  =  fitted lognormal distributions. HS  =  Huangshan students, SS  =  Shenzhen students, HE  =  Huangshan employees, SE  =  Shenzhen employees, m =  male, f =  female, urb  =  urban area, rur  =  rural area, n reg  =  not registered, reg  =  registered.

We fitted lognormal distributions to the distance distributions stratified by study area and employment status, and further into the categories determined by the linear regression. Cumulative observed and fitted lognormal distance distributions are shown in Figure 6 showing a reasonable agreement between data and fit, see Table S5 to Table S8 in Text S2 for the fitted parameter values and goodness of fit statistics.

We also fitted several other distributional forms, including exponential, power law and Weibull distributions, to the commuting distance distributions, however, the lognormal tended to give the best fit for the majority of strata.

### Occasional travel outside the study area

The mean number of journeys undertaken outside the study area within the 7 days prior to the questionnaire being taken varied markedly between the different study populations, see Table 2, with those in urban Huangshan travelling much more frequently than any other group. However, one should keep in mind that the study area of urban Huangshan is very small, and therefore many shorter journeys are counted, which in any other study area would remain within the area and therefore not be taken into account. While the majority of people did not travel at all, the number of people having made more than one journey is higher than would be expected if all people travelled with the same probability (p = 1.7·10^−8^, p = 6·10^−5^, p = 0.015, for urban Huangshan, rural Huangshan and Shenzhen, respectively), indicating inhomogeneity in travelling behaviour – i.e. the majority of travelling is done by a minority of the population (see Text S2 for details). However, it should be noted that the same pattern could be observed if the journeys an individual undertakes are clustered in time.

**Table 2:** Number of journeys undertaken in the past seven days for the different study populations.

Number of journeys	urban Huangshan	rural Huangshan	Shenzhen local	Shenzhen migrant	Total
number of people travelling	224	233	360	30	847
total number of journeys	286	253	380	31	950
mean number of journeys	0.12	0.031	0.038	0.016	0.043

The best fit logistic regression model for predicting who had made at least one journey outside the study area for the Huangshan city population included the following covariates: rural/urban area, sex, distance travelled to work or school, and occupation. People from the urban area, males, those with a long commute (> = 10 km) to work or school travelled more, and those who were unemployed travelled less than either students or employees. All included variables are highly significant, apart from the lack of differentiation between students and employees.

For Shenzhen city, the best fit logistic regression model included registration status, distance to work or school, subpopulation (local or migrant workers) and family size. Those registered, with a long distance to work/school, from the local population and from smaller families travelled most, with all variables being highly significant. Odds ratios for these models are shown in Table 3 and Table 4.

**Table 3:** Results of the logistic regression analysis for having travelled within the past 7 days for Huangshan.

		N	% travelled	OR (95% CI)	p
area	urban	2089	9.68		
	rural	7865	2.88	0.24 (0.19–0.30)	<5e-4
sex	male	4877	5.43		
	female	5077	3.37	0.65 (0.54–0.78)	<5e-4
distance to work/school	<10 km	8621	4.05		
	> = 10 km	1333	6.52	1.5 (1.2–2.0)	<5e-4
occupation	student	1567	5.89		
	employee	6673	4.47	0.93 (0.73–1.17)	0.527
	unempl	1714	2.67	0.41 (0.28–0.59)	<5e-4

**Table 4:** Results of the logistic regression analysis for having travelled within the past 7 days for Shenzhen.

		N	% travelled	OR (95% CI)	p
registration	not reg	9789	2.53		
	reg	2091	6.79	2.5 (1.9–3.3)	<5e-4
distance to work/school	<10 km	10962	2.98		
	> = 10 km	918	6.86	1.9 (1.4–2.6)	<5e-4
subpopulation	local	9886	3.64		
	migrant	1994	1.50	0.47 (0.31–0.71)	<5e-4
family size	< = 3	6801	3.44		
	>3	5079	3.07	0.68 (0.51–0.91)	0.011

**Figure 7:**
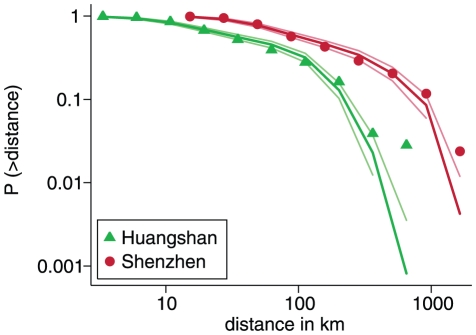
Observed and fitted cumulative distributions of travel distances for Huangshan and Shenzhen. Symbols  =  observed distributions, thick lines  =  fitted distributions, pale lines  =  95% credibility intervals of the fitted distributions.

We fitted a simplified gravity model with a lognormal kernel function to the observed distances travelled. The lognormal distribution yielded a better fit than other distributions, namely exponential, power law and Weibull. Figure 7 shows a plot of the observed cumulative distance distributions for Huangshan and Shenzhen compared to the fitted distributions. Although the observed distributions are sometimes outside the 95% credibility interval, the overall fit is quite convincing. The fitted parameters with confidence intervals are shown in Table 5. The travel distances observed in Huangshan are shorter than those in Shenzhen, which is reflected in the difference in parameter estimates for the log median distance 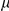. This could be explained by a differential in wealth between Huangshan and Shenzhen. The estimates of the standard deviation of the log distance are very similar for Huangshan and Shenzhen.

**Table 5:** Parameters and 95% confidence intervals of the gravity model fitted to the travel distance distributions.

	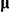	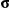
Huangshan	2.70 (2.55–2.84)	1.04 (0.98–1.12)
Shenzhen	4.85 (4.70–4.99)	1.21 (1.09–1.35)

## Discussion

We have analysed a unique dataset of commuting and travel behaviour in two very different parts of China. The commuting and travel patterns show interesting differences between these two study sites, indicating that there is substantial heterogeneity in China. This means that it is not easy to generalise our results to the whole country, although the overall demographics of China resemble more those in Huangshan, whereas the strongly peaked age distribution found in Shenzhen that reflects the large number of migrant workers appears to be fairly unique (see Text S2).

Study participants were chosen by household. In Huangshan, households were selected from one of the urban districts and one of the rural counties to match the overall proportion of urban and rural residents, whereas in Shenzhen, households were chosen from the local residents population and the dormitories housing the migrant workers. This selection process should give a fair representation of the overall populations in Huangshan and Shenzhen, respectively, as long as these are the major subpopulations. However, should there be substantial differences between the different urban districts and rural counties in Huangshan or any other important population groups in Shenzhen that are not covered in the selection of households, it is possible that the study populations might not reflect the cities well.

In China, the hukou system of residency permit [12], [13] controls the influx of people from rural areas into the cities, where wages are higher and access to education and other services is better. It is therefore not surprising that in the rural area of Huangshan virtually everyone has a residency permit, whereas in the Shenzhen local resident population, the proportion with a residency permit is low, and less than 1% of the migrant workers have a residency permit.

Migrant workers move from rural areas to the cities in order to take up jobs in manufacturing for at least 6 months of the year, living in company provided dormitories. The fact that around 17% of Shenzhen's population is made up of these migrant workers points to the rapid growth of the local economy and its need for labour as well as the differential in wages between Shenzhen and other areas of China. The dormitories provide very crowded accommodation with typically 8–12 people sleeping in the same room and many more assemble in the communal areas for eating and other pursuits [14]. While these dormitories are a potential focus of disease spread, the commuting distances (up to 7 km) for the migrant workers are the shortest observed in all datasets. We would therefore not expect this population to play a major role in the spatial spread of acute disease (such as influenza) themselves, but only via contact to other parts of the population who have longer commuting distances. However, if the migrants travel to their home provinces on a seasonal basis (which was not detected in this survey), they might indeed play a larger role in the long distance spread of chronic infectious diseases such as HIV or TB.

The regression modelling has identified several predictors of the distance to school or work, but the best fitting models explain only a small part of the observed variability, pointing to probably a large number of factors that are not well understood influencing the complex choices of places to live and work. However, the covariates included in the models do indicate that different mechanisms determine the distance to school or work for students and employees.

For students, the most important factor is student age, with older students travelling further as the number of schools decreases from primary to secondary schools. As the population density is lower in the rural areas, students in rural Huangshan tend to have to traverse longer distances to get to their school than those in the urban area. In Shenzhen, students without residency permit tend to have longer distances to school, possibly reflecting the more difficult access to education for the unregistered population [15].

For employees, it is tempting to surmise that a long commuting distance is associated with a higher socio-economic status, as has been reported for other places such as Seoul [16]. Certainly we find in Shenzhen that those with residency permits have larger commuting distances, whereas the shortest distances are found in the Shenzhen migrant worker population. In Huangshan, commuting distances tend to be larger in the rural area. The dependence of commuting distance on age, with shorter distances for older people, is interesting and could be explained by a number of mechanisms. For instance, it is possible that over long timescales people change jobs or move if a more convenient opportunity arises, and that in this process the commuting distances tend to settle down to a more local distribution. It is also conceivable that over time there has been a shift in attitudes with younger people being more accepting of longer commuting distances.

These mobility determinants might interact in interesting ways with other characteristics important for disease spread, such as, for instance, an interaction between the age-dependence of commuting distance with the age-dependence of contact patterns or biological susceptibility to disease.

Travelling behaviour does not differ substantially between students and employees, but otherwise the predictors of travelling are similar to those for long commuting distances, with a long commuting distance being explicitly included in the logistic regression models for travelling.

**Figure 8:**
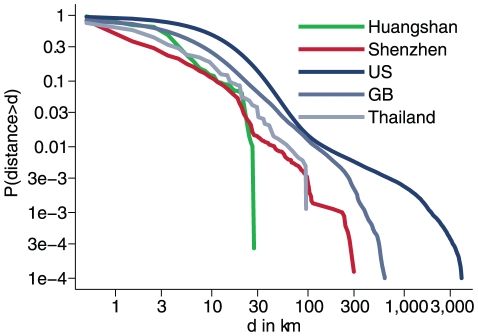
Commuting distance distributions for employees in the study population compared to those from other countries.

Viboud et al. [4] found that commuting flows predict the spatial spread of seasonal flu in the US. As people travelling long distances on a regular basis would be expected to contribute most to the spatial spread, the tail of the distance distribution has most impact on the potential spread of diseases. The distributions of commuting distances for both Shenzhen and Huangshan are considerably more local than those from western counties (UK, US [17], [18], see Figure 8), where power laws or exponential distributions have been used to model distance distributions [4], [8], [10], [19]. There are however massive differences between the two Chinese cities with the distance distribution for Huangshan city being very local, with no one in our datasets commuting further than 30 km, whereas the distribution found in Shenzhen is comparable to, albeit a little more local than that from Thailand [6]. Possible reasons for these differences include a weaker infrastructure or affordability of travelling. The hukou system, which officially requires people to work and live where they are registered, could also explain much of the locality seen in commuting patterns.

While the patterns of occasional journeys observed might have a smaller impact on disease spread than commuting distances, due to the lower frequency of journeys, infrequent but very long distance trips are likely to play an important role in spreading diseases between regions, particularly in areas like Huangshan, where commuting is limited to very short distances.

This paper has presented the first known data on human travel patterns within China. The striking differences between travel distances seen in Huangshan and Shenzhen point to substantial heterogeneity in travel behaviour within China at its current state of development. It is important to take these regional differences into account when modelling the likely speed of geographic spread of an infectious disease outbreak and in planning for containment or control of such outbreaks. Ongoing modelling work is using these data to examine the feasibility of containment of a lethal influenza pandemic in different areas of China.

## Supporting Information

Text S1 **Questionnaires.**
(PDF)

Text S2 **Additional Detail of the Analyses.**
(PDF)
