# Correction: Travel Patterns in China

**DOI:** 10.1371/annotation/e4781265-2e4f-45a9-85b7-1930e0a16171

**Published:** 2011-02-09

**Authors:** Tini Garske, Hongjie Yu, Zhibin Peng, Min Ye, Hang Zhou, Xiaowen Cheng, Jiabing Wu, Neil Ferguson

Equal contribution was unassigned. Tini Garske and Hongjie Yu contributed equally to this work. 

