# Supplementary material for: Travel Patterns in China
Source: PLoS One. 2011 Feb 2;6(2):e16364. doi: 10.1371/journal.pone.0016364 (PMC3032737; doi:10.1371/journal.pone.0016364)
Supplement: Text S1 — Questionnaires. (PDF) [file pone.0016364.s001.pdf]

Travel Patterns in China

Questionnaires

Tini Garske[1†], Hongjie Yu[2*], Zhibin Peng[2], Min Ye[2], Hang Zhou[2], Xiaowen Cheng[3], Jiabing Wu[4], Neil Ferguson[1]*

[1]MRC Centre of Outbreak Analysis and Modelling, Imperial College London

[2]Office for Disease Control and Emergency Response, Chinese Center for Disease Control and Prevention (China CDC), Beijing, China

[3] Department of Communicable Disease Prevention and Control, Shenzhen Center for Disease Control and Prevention, Shenzhen, China

[4] Department of Communicable Disease Prevention and Control, Anhui Provincial Centers for Disease Control and Prevention, Hefei, China

[†]Email: t.garske@imperial.ac.uk

[*]Contributed equally

Registration form (resident traveling survey)

____Prefecture____District/County____Community/ factory /village Association   Page：

Date：______________ (mm/dd/year)   Investigator：

ID No.	Home Address	No response reason for household [1]	No response reason for household member[2]							
			1[3]	2	3	4	5	6	7	8

Note:

1. ① Nobody at home, ② Refuse to participate, ③ Other (if choose ③, please write down the reason directly in the blank)
2. ① Refuse to participate, ② Other (if choose ②, please write down the reason directly in the blank)
3. The Arabic numerals are ID No. for each family member, which must be consistent with that in the questionnaire.

Questionnaire ---- Shenzhen

Resident Traveling Survey

Good morning/afternoon/evening !

With the spread of H5N1, potential next pandemic will be a terrible threat to human beings. It is necessary for us to develop feasible and effective pandemic preparedness and response strategy. The resident traveling survey is implemented by Chinese Center for Disease Control and Prevention. The purpose of the survey is to learn about the residents' traveling habits through collecting individual traveling data (including the distance from home to work/school and other traveling within past 7 days) to provide scientific evidence for establishing pandemic modeling and developing the strategy of pandemic influenza preparedness and response in China.

We mainly collect the information about your everyday traveling such as going to work or school and other traveling within past 7 days. Simple demographic data such as gender and age will also be collected. There are not any sensitive or private questions in the questionnaire, so you will not feel any discomforts. Meanwhile we will not begin the face to face survey until obtain oral informed consent from you. We promise that all the information you provide will be kept secret strictly. You will be voluntary for the survey. If you are not willing to take part in our survey, it would not bring any bad impact for you. It will take you less than 10 minutes to finish the survey, we feel sorry for bothering you. If you have any question, please consult our investigators, we will try our best to explain for you.

If you consent to participate, let's begin the survey. Please answer all the questions truthfully. We really appreciate you for your kindly help. Thank you very much!

Sincerely

Verbal informed consent _______①yes, ②**no** **Date:__________(mm/dd/year)**

Chinese Center for Disease Control and Prevention

2007-7

Resident Traveling Survey Questionnaire

ID Number：____________

Investigator:_____ **Interview Date: ____(mm/dd/year)**

Checker:_________ **Check Date: _______(mm/dd/year)**

Home Address: _Shenzhen__Prefecture ____District___Township_______Community

Section One Distance travel to workplace/school

No.[1]	Age	Sex	Vocation[2]	Household Registry[3]	Dormitory[4]	GPS Distance (km)	Estimated Distance[5] (km)	out of mainland China[6]	All other travel outside of local area in the last 7 days[7]	comment
1										
2										
3										
4										
5										
6										
7										
8										

Note：

1. Every family member should be interviewed, the information should get form his/he family members if the participant is not available when interviewing, and **the sequence should be** consistent with section two.
2. ①student ②working ③non-working
3. ①Residents with local Household Register ②Residents with no local Household Register and living ≥ 3 months ③Residents with no local Household Register and living < 3 months, ④Residents with no local Household Register and living < 2 months, ⑤Residents with no local Household Register and living < 1 months.

4. If the interviewee lived in the factory dormitories, please mark "√" in the blank.

5. If the distance can't be measured by GPS, the interviewees have to estimate the distance by themselves (kilometer is used as the unit for distance value which needs to be rounded off to one decimal).

6. If the interviewee's workplace or school is located out of mainland China, please fill the location in the blank (HK SAR , Macao etc).

7. If the interviewee has other travel outside of local area in the last 7 days, please mark "√" in the blank. (Shenzhen：the journey out of Shenzhen)

8. Fill in the average number of contacts in the last 7 days of each individual. level 1: physical contact without conversation; level 2: conversation without physical contact; level 3: conversation with physical contact.

Section Two All other travel outside of local area in the last 7 days[1]

No.[2]	Times	National					International				
		Destination[3]	GPS Distance[4] (km)	Urban To rural[5]	Rural to urban[5]	Travel Reason[6]	Destination[3]			Distance[7] (km)	Travel Reason[6]
		__Province Prefecture____District/County					HK &Macao[5]	Other[5]			
								__Country__Province			
	①										
	②										
	③										
	①										
	②										
	③										
	①										
	②										
	③										
	①										
	②										
	③										
	①										
	②										
	③										

Note：

1. This part is set aside only for the interviewees who has other travel outside of local area in the last 7 days.
2. Fill the ID No. of the interviewees who has journeys caused by non-work/non-school in the last 7 days. and **the sequence should be** consistent with section one.
3. If the destinations are more than one place, please fill all the destinations in the blank.
4. If the destinations are more than one place, please fill all the GPS distance in the blank.

5. For national travel, if the journey includes flows between rural area and urban area, please mark "√" in the blank. For international travel, if the destination is HK SAR, please fill①in **"HK&Macao "** column, if the destination is Macao, please fill②in **"HK&Macao "** column, if the destinations include other countries besides or except **HK&Macao**, please fill the actual address in **Other** column.(Other: all other countries except HK SAR and mainland China).
6. ①Business ②Tour ③Visit relatives and friends ④Other (If choose ④, write down travel reason directly in the blank).
7. **Don't need to fill.**

Questionnaire ---- Huangshan

Resident Traveling Survey

Good morning/afternoon/evening !

With the spread of H5N1, potential next pandemic will be a terrible threat to human beings. It is necessary for us to develop feasible and effective pandemic preparedness and response strategy. The resident traveling survey is implemented by Chinese Center for Disease Control and Prevention. The purpose of the survey is to learn about the residents' traveling habits through collecting individual traveling data (including the distance from home to work/school and other traveling within past 7 days) to provide scientific evidence for establishing pandemic modeling and developing the strategy of pandemic influenza preparedness and response in China.

We mainly collect the information about your everyday traveling such as going to work or school and other traveling within past 7 days. Simple demographic data such as gender and age will also be collected. There are not any sensitive or private questions in the questionnaire, so you will not feel any discomforts. Meanwhile we will not begin the face to face survey until obtain oral informed consent from you. We promise that all the information you provide will be kept secret strictly. You will be voluntary for the survey. If you are not willing to take part in our survey, it would not bring any bad impact for you. It will take you less than 10 minutes to finish the survey, we feel sorry for bothering you. If you have any question, please consult our investigators, we will try our best to explain for you.

If you consent to participate, let's begin the survey. Please answer all the questions faithfully. We really appreciate you for your kindly help. Thank you very much!

Sincerely

Verbal informed consent ______①yes, ②no **Date:__________(mm/dd/year)**

Chinese Center for Disease Control and Prevention

2007-7

Resident Traveling Survey Questionnaire

ID Number: ____________

Investigator: __________ **Interview Date: ______________ (mm/dd/year)**

Checker: _________ **Check Date: _______________(mm/dd/year)**

**HomeAddress: _ Huangshan _Prefecture ______District/County_________Township_______Community/ Village Street/Road**							
**Section One Distance travel to workplace/school**							
**No.** [1]	**Age**	**Sex**	**Vocation** [2]	**Household Registry** [3]	**Estimated Distance(km)** [4]	**All other travel outside of local area in the last 7 days** [5]	**Comment**
1							
2							
3							
4							
5							
6							
7							
8							

Note:

1. Every family member should be interviewed, the information should get form his/he family members if the participant is not available when interviewing, and **the sequence should be** consistent with section two.

2. ① student, ② working, ③ non-working
3. ① Residents with local Household Register, ② residents with no local Household Register and living ≥ 3 months, ③ residents with no local Household Register and living < 3 months, ④ Residents with no local Household Register and living < 2 months, ⑤ Residents with no local Household Register and living < 1 months.
4. Estimated by the interviewees themselves, kilometer is used as the unit for distance value which needs to be rounded off to one decimal.
5. If the interviewee has other travel outside of local area, please mark "√" in the blank. (Tunxi District: the journey out of Tunxi District, Xiuning County: the journey out of Xiuning County).

Section Two All other travel outside of local area in the last 7 days [1]											
No. [2]	Times	National					International				
		Destination [3]<br>__Province<br>Prefecture____District/County	GPS Distance[4] (km)	Urban To rural [5]	Rural to urban[5]	Travel Reason [6]	Destination [3]<br>HK &Macao [5]	Other [5]<br>__Country__Province		Distance[4] (km)	Travel Reason [6]
	①										
	②										
	③										
	①										
	②										
	③										
	①										
	②										
	③										
	①										
	②										
	③										
	①										
	②										
	③										

Note:

1. This part is set aside only for the interviewees who has other travel outside of local area in the last 7 days.
2. Fill the ID No. of the interviewees who has their travel outside of local area in the last 7 days. and **the sequence should be** consistent with section one.
3. If the destinations are more than one place, please fill all the destinations in the blank.
4. **Don’t need to fill.**

5. For national travel, if the journey includes flows between rural area and urban area, please mark "√" in the blank. For international travel, if the destination is HK SAR, please fill①in **"HK&Macao "** column, if the destination is Macao, please fill ② in **"HK&Macao "** column, if the destinations include other countries besides or except **HK&Macao**, please fill the actual address in **Other** column.(Other: all other countries except HK SAR and mainland China).
6. ① Business, ② Tour, ③ Visit relatives and friends, ④ Other (If choose ④, write down travel reason directly in the blank)
