# Supplementary material for: Travel Patterns in China
Source: PLoS One. 2011 Feb 2;6(2):e16364. doi: 10.1371/journal.pone.0016364 (PMC3032737; doi:10.1371/journal.pone.0016364)
Supplement: Text S2 — Additional Detail of the Analyses. (PDF) [file pone.0016364.s002.pdf]

Travel Patterns in China
Additional Detail of the Analyses

Tini Garske[1†], Hongjie Yu[2*], Zhibin Peng[2], Min Ye[2], Hang Zhou[2], Xiaowen Cheng[3], Jiabing Wu[4], Neil Ferguson[1]*

[1]MRC Centre of Outbreak Analysis and Modelling, Imperial College London
[2]Office for Disease Control and Emergency Response, Chinese Center for Disease Control and Prevention (China CDC), Beijing, China
[3] Department of Communicable Disease Prevention and Control, Shenzhen Center for Disease Control and Prevention, Shenzhen, China
[4] Department of Communicable Disease Prevention and Control, Anhui Provincial Centers for Disease Control and Prevention, Hefei, China

[†]Email: t.garske@imperial.ac.uk

[*]Contributed equally

Demographics

The population census 2000 provides age distributions in 5 year age bands by administrative area. Figure S1a shows the age distributions of Shenzhen and Huangshan compared with that of China overall. Most remarkable is the large proportion of people in their 20s in Shenzhen, clearly indicating the population of migrant workers (cf. Figure 3 in the main text). It is also clear that the Chinese population is demographically not stable. This could be due to the One Child Policy implemented in 1979, and other factors [1]. Due to the rapid changes in the demography there is little merit in comparing the age distributions found in the study populations in 2007 with the population census data from 2000 directly. In order to make the data more comparable, we project the age distribution of the study populations back to the year 2000 by subtracting 7 years from each individual's age and re-normalising the distributions. While this is the best approximation to the actual age distribution the study population would have had in 2000, it does not account for any deaths that would have occurred between 2000 and 2007 (hence the proportion of the elderly tends to be lower in the back projected distributions than in the census), and for any migration patterns. **Figure S1** b to d compare the age distributions from the census in 2000 with those of the study populations projected back to the year 2000 for urban and rural Huangshan and Shenzhen, respectively. In Huangshan, the distributions match fairly well for adults over about 35 years, but differ markedly for younger people. This may be due to migration of the younger population. For Shenzhen, we compare both the back projected and the current age distribution of the study population with the census data. In the study population, the peak in the age distribution is less pronounced than that in the population census, and interestingly the peak age (20-24 years) of the current (rather than the back projected) study population matches that of the population census in 2000. This might be an indication of a high turnover of the migrant worker population, whereas the broadening of the peak would be explained by the ageing of the more stable local residents population.

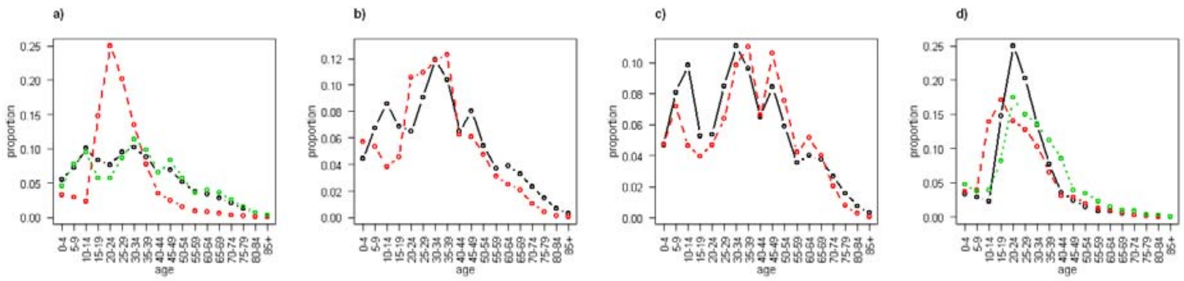

Figure S1: Age distributions in China. a) China (solid black line), Shenzhen (long dashed red line) and Huangshan (dashed green line) from the population census 2000. b) Urban Huangshan from the population census 2000 (solid black line) and urban Huangshan study population projected back to 2000 (long dashed red line). c) Rural Huangshan from the population census 2000 (solid black line) and rural Huangshan study population projected back to 2000 (long dashed red line). d) Shenzhen from the population census 2000 (solid black line), Shenzhen study population projected back to 2000 (long dashed red line) and in 2007 (dashed green line).

In China, employment rates rise rapidly between ages 15 and 20 for both men and women. Between 20 and 50 years, employment is at its highest level, with around 95% of men, but only 85% of women employed. After the age of 50, employment rates start to decline as people retire. However, there are marked differences between regions. Both the urban study areas mirror the lower employment rate for women with particularly low employment among Shenzhen women from their mid-twenties onwards. Retirement age starts earlier than the national average. Rural Huangshan shows very high employment rates (close to 100%) for both men and women and a much higher retirement age, see Figure S2.

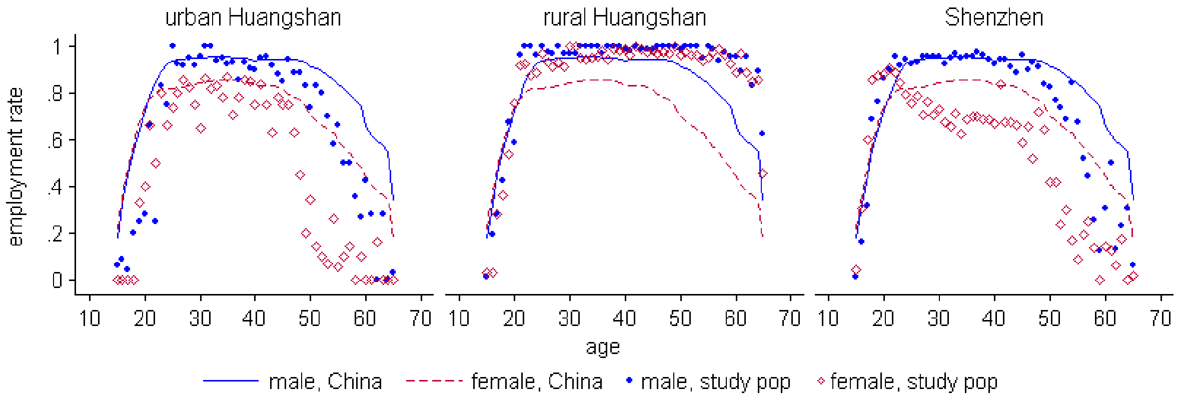

Figure S2: Employment rates for men and women in the study populations compared to the overall employment rates for China.

The proportion of the study populations in education is shown in Figure S3. Between the ages of 6 and 15, nearly full enrolment is achieved. Between 0 and 5 years, enrolment rates rise rapidly; urban Huangshan has considerably higher enrolment rates in both very young children and older teenagers than either rural Huangshan or Shenzhen.

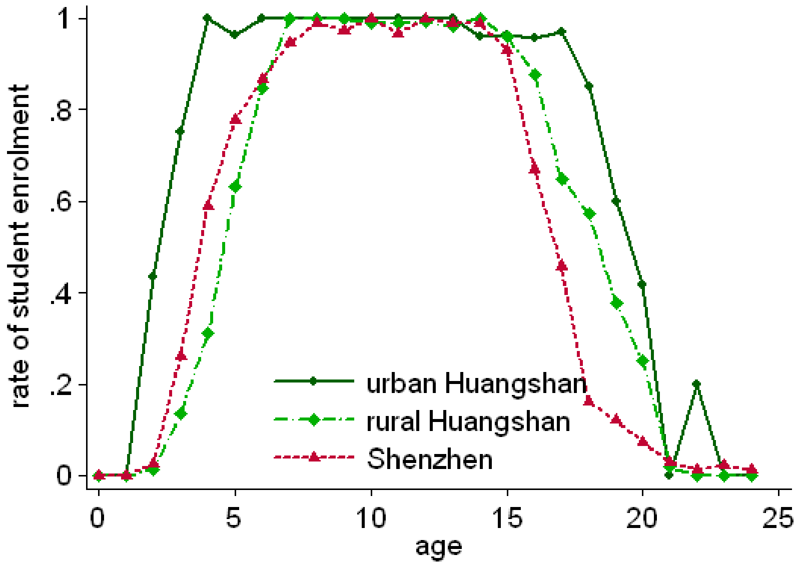

Figure S3: Student enrolment rates for the different study populations.

Additional detail on the regression analysis

For Huangshan students, the best fitting model includes age and urban/rural, with an interaction. The fitted mean log commuting distance is therefore given by

$$\widehat{\ln d} = A + B_{\text{age}} + C_{\text{urb/rur}} + D_{\text{age*urb/rur}} \,. \tag{S1}$$

Note that $B_{0-5} = C_{\text{urb}} = D_{\text{age*urb}} = D_{0-5\text{*rur}} = 0$ by definition. For Shenzhen students, the best fitting model includes age and registration status, without an interaction term, such that the fitted mean log commuting distance is given by

$$\widehat{\ln d} = A + B_{\text{age}} + C_{\text{reg stat}} \,, \tag{S2}$$

where $B_{0-5} = C_{\text{n reg}} = 0$.

For Huangshan employees, the best fitting model includes age, sex and urban/rural with no interactions, and we have

$$\widehat{\ln d} = A + B_{\text{age}} + C_{\text{urb/rur}} + D_{\text{sex}} \tag{S3}$$

with $B_{0-19} = C_{\text{urb}} = D_{\text{male}} = 0$. For Shenzhen employees, the best fitting model includes age, registration status and sex with interactions between age and sex and between registration status and sex. Furthermore, migrant workers are included as a separate category. For Shenzhen local employees, the fitted mean log distance is therefore given by

$$\widehat{\ln d} = A + B_{\text{age}} + C_{\text{reg}} + D_{\text{sex}} + E_{\text{age*sex}} + F_{\text{reg stat*sex}} \,, \tag{S4}$$

with $B_{0-19} = C_{\text{n reg}} = D_{\text{male}} = E_{\text{age*male}} = E_{\text{0-19*female}} = F_{\text{n reg*}sex} = F_{\text{reg*male}} = 0$. The non-zero parameters for these four models are displayed in Table S1 to Table S4. As expected, the p-values for most parameters are highly significant.

Furthermore, we fitted lognormal distributions to the observed distances stratified by the categories identified in the regression analysis. The parameter values of these are given in Table S5 to Table S8. Although the fitted distributions shown in Figure 6 in the main text are visually quite convincing, the p-values for many strata are very small, indicating a significant deviation of the observed from the fitted distributions. This is particularly the case for the both Huangshan students and employees, as the sharp cut-off around 30km in the distributions is difficult to capture.

Table S1: Fitted parameters (95% CI) and p-values of the best fitting linear regression models for Huangshan students.

	coefficient	p
A	0.36 (0.20 - 0.51)	<0.01
B_{6-11}	0.33 (0.13 - 0.52)	<0.01
B_{12-14}	0.35 (0.10 - 0.60)	<0.01
B_{15-17}	0.99 (0.76 - 1.23)	<0.01
B_{18+}	1.3 (0.9 - 1.6)	<0.01
C_{rur}	0.9 (0.6 - 1.2)	<0.01
$D_{6-11*rur}$	-0.3 (-0.7 - 0.0)	0.08
$D_{12-14*rur}$	0.4 (0.0 - 0.8)	0.06
$D_{15-17*rur}$	0.4 (0.1 - 0.8)	0.03
$D_{18+*rur}$	0.2 (-0.2 - 0.7)	0.31

Table S2: Fitted parameters (95% CI) and p-values of the best fitting linear regression models for Shenzhen students.

	coefficient	p
A	0.54 (0.34 - 0.74)	<0.01
B_{6-11}	0.18 (-0.06 - 0.41)	0.14
B_{12-14}	0.5 (0.2 - 0.8)	<0.01
B_{15-17}	0.9 (0.6 - 1.2)	<0.01
B_{18+}	2.1 (1.7 - 2.4)	<0.01
C_{reg}	-0.34 (-0.54 - -0.14)	<0.01

Table S3: Fitted parameters (95% CI) and p-values of the best fitting linear regression models for Huangshan employees.

	coefficient	p
A	1.60 (1.38 - 1.82)	<0.01
B_{20-29}	-0.22 (-0.43 - -0.01)	0.04
B_{30-39}	-0.43 (-0.64 - -0.23)	<0.01
B_{40-49}	-0.55 (-0.75 - -0.35)	<0.01
B_{50-59}	-0.67 (-0.87 - -0.47)	<0.01
B_{60+}	-0.82 (-1.03 - -0.62)	<0.01
C_{rur}	0.19 (0.08 - 0.30)	<0.01
D_{female}	-0.21 (-0.25 - -0.17)	<0.01

Table S4: Fitted parameters (95% CI) and p-values of the best fitting linear regression models for Shenzhen employees.

	coefficient	p
A	-0.02 (-0.26 - 0.22)	0.87
B_{20-29}	0.6 (0.3 - 0.8)	<0.01
B_{30-39}	0.73 (0.47 - 0.99)	<0.01
B_{40-49}	0.6 (0.3 - 0.9)	<0.01
B_{50-59}	0.4 (0.1 - 0.7)	0.01
B_{60+}	-0.2 (-1.0 - 0.5)	0.49
C_{reg}	0.68 (0.53 - 0.82)	<0.01
D_{female}	0.2 (-0.1 - 0.5)	0.25
$E_{20-29*female}$	-0.4 (-0.7 - 0.0)	0.03
$E_{30-39*female}$	-0.9 (-1.2 - -0.5)	<0.01
$E_{40-49*female}$	-1.0 (-1.3 - -0.6)	<0.01
$E_{50-59*female}$	-1.2 (-1.7 - -0.7)	<0.01
$E_{60+*female}$	-0.4 (-1.3 - 0.5)	0.40
$F_{reg*female}$	0.43 (0.24 - 0.62)	<0.01

Table S5: Number of distances, number of distances used in the fit, parameter values (95% CIs) for the lognormal distributions and p-values for the overall goodness of fit for to the different strata of the commuting distance distributions for Huangshan students.

urb/rur	age	N	N>1.45	μ	σ	excess <1.45km	p
urban	0-5	89	27	1.2 (0.5 - 1.4)	0.48 (0.32 - 0.96)	0.683 (0.447 - 0.696)	0.35
urban	6-11	158	76	1.30 (1.03 - 1.48)	0.56 (0.44 - 0.78)	0.495 (0.406 - 0.514)	<0.01
urban	12-14	73	40	1.31 (0.90 - 1.53)	0.53 (0.38 - 0.86)	0.431 (0.260 - 0.450)	<0.01
urban	15-17	81	66	1.62 (1.45 - 1.78)	0.50 (0.40 - 0.66)	0.180 (0.150 - 0.185)	<0.01
urban	18+	38	32	1.9 (1.5 - 2.2)	0.62 (0.46 - 0.96)	0.151 (0.070 - 0.158)	<0.01
rural	0-5	78	47	2.1 (1.7 - 2.4)	0.80 (0.61 - 1.15)	0.388 (0.327 - 0.396)	<0.01
rural	6-11	441	292	1.90 (1.81 - 1.99)	0.60 (0.54 - 0.68)	0.334 (0.329 - 0.337)	<0.01
rural	12-14	351	307	2.26 (2.16 - 2.34)	0.63 (0.57 - 0.70)	0.1241 (0.1217 - 0.1250)	<0.01
rural	15-17	269	266	2.71 (2.62 - 2.81)	0.61 (0.55 - 0.69)	0.01109 (0.01080 - 0.01114)	<0.01
rural	18+	100	100	2.79 (2.61 - 2.98)	0.74 (0.63 - 0.90)	-0.0005 (-0.0042 - 0.0000)	<0.01

Table S6: Number of distances, number of distances used in the fit, parameter values (95% CIs) for the lognormal distributions and p-values for the overall goodness of fit for to the different strata of the commuting distance distributions for Shenzhen students.

reg stat	age	N	N>1.45	μ	σ	excess <1.45km	p
not reg	0-5	114	51	-13 (-2086 - 0)	4.3 (1.6 - 58.1)	-499 (-∞ - 0)	<0.01
not reg	6-11	369	181	-18 (-2224 - -2)	5.5 (2.5 - 60.3)	-1270 (-∞ - -1)	<0.01
not reg	12-14	183	101	-6 (-2894 - 1)	3.9 (2.1 - 78.2)	-7.5 (-∞ - 0.0)	<0.01
not reg	15-17	127	80	-7 (-3369 - 1)	5 (2 - 92)	-11 (-∞ - 0)	<0.01
not reg	18+	55	49	2.7 (0.6 - 3.5)	1.9 (1.4 - 3.2)	0.01 (-0.69 - 0.09)	<0.01
reg	0-5	50	16	1.3 (-38.5 - 1.8)	0.7 (0.4 - 6.6)	0.65 (-∞ - 0.68)	1.00
reg	6-11	133	54	-0.6 (-1568.5 - 1.2)	1.7 (1.0 - 45.0)	-0.46 (-∞ - 0.49)	0.01
reg	12-14	86	53	-0.4 (-1830.0 - 1.4)	1.8 (1.0 - 51.6)	-0.8 (-∞ - 0.2)	0.08
reg	15-17	108	71	0.9 (-6.4 - 1.7)	1.5 (1.0 - 3.7)	0.0 (-24.6 - 0.3)	0.68
reg	18+	107	93	2.4 (1.8 - 2.8)	1.33 (1.06 - 1.79)	0.07 (-0.08 - 0.12)	<0.01

Table S7: Number of distances, number of distances used in the fit, parameter values (95% CIs) for the lognormal distributions and p-values for the overall goodness of fit for to the different strata of the commuting distance distributions for Huangshan employees.

urb/rur	sex	age	N	N>1.45	μ	σ	excess <1.45km	p
urban	m	0-19	7	2	(-)	(-)	(-)	
urban	m	20-29	89	72	1.6 (0.9 - 2.0)	1.0 (0.7 - 1.4)	0.10 (-0.24 - 0.17)	<0.01
urban	m	30-39	231	166	1.75 (1.51 - 1.93)	0.85 (0.72 - 1.05)	0.24 (0.17 - 0.27)	<0.01
urban	m	40-49	213	154	1.84 (1.61 - 2.02)	0.83 (0.70 - 1.02)	0.25 (0.19 - 0.27)	<0.01
urban	m	50-59	85	58	1.5 (0.7 - 1.9)	0.89 (0.66 - 1.42)	0.24 (-0.15 - 0.30)	<0.01
urban	m	60+	10	7	2.0 (0.5 - 2.7)	0.6 (0.3 - 1.8)	0.2989 (-0.3453 - 0.3000)	0.25
urban	f	0-19	2	2	(-)	(-)	(-)	
urban	f	20-29	117	88	1.5 (0.9 - 1.8)	0.88 (0.68 - 1.26)	0.16 (-0.12 - 0.22)	<0.01
urban	f	30-39	227	154	1.55 (1.27 - 1.74)	0.81 (0.67 - 1.02)	0.27 (0.17 - 0.30)	<0.01
urban	f	40-49	157	90	1.59 (1.32 - 1.79)	0.69 (0.56 - 0.92)	0.404 (0.336 - 0.421)	<0.01
urban	f	50-59	21	13	1.3 (-1490.7 - 1.8)	0.7 (0.4 - 43.7)	0.32 ($-\infty$ - 0.38)	0.02
urban	f	60+	1	0	(-)	(-)	(-)	
rural	m	0-19	74	60	2.1 (1.8 - 2.4)	0.75 (0.60 - 1.02)	0.181 (0.136 - 0.188)	<0.01
rural	m	20-29	289	236	1.98 (1.82 - 2.11)	0.82 (0.71 - 0.95)	0.163 (0.134 - 0.175)	<0.01
rural	m	30-39	553	417	1.87 (1.75 - 1.98)	0.82 (0.73 - 0.92)	0.220 (0.197 - 0.233)	<0.01
rural	m	40-49	688	511	1.62 (1.50 - 1.73)	0.81 (0.73 - 0.91)	0.209 (0.172 - 0.230)	<0.01
rural	m	50-59	799	568	1.43 (1.34 - 1.51)	0.64 (0.58 - 0.71)	0.252 (0.226 - 0.268)	<0.01
rural	m	60+	546	361	1.31 (1.21 - 1.40)	0.58 (0.51 - 0.66)	0.303 (0.267 - 0.321)	<0.01
rural	f	0-19	46	43	1.9 (1.2 - 2.2)	0.88 (0.65 - 1.41)	0.02 (-0.28 - 0.06)	<0.01
rural	f	20-29	340	254	1.72 (1.55 - 1.86)	0.80 (0.70 - 0.94)	0.217 (0.173 - 0.237)	<0.01
rural	f	30-39	564	384	1.58 (1.46 - 1.69)	0.72 (0.64 - 0.82)	0.286 (0.254 - 0.302)	<0.01
rural	f	40-49	740	527	1.35 (1.26 - 1.43)	0.61 (0.55 - 0.68)	0.249 (0.218 - 0.266)	<0.01
rural	f	50-59	755	530	1.38 (1.31 - 1.44)	0.53 (0.48 - 0.58)	0.278 (0.261 - 0.287)	<0.01
rural	f	60+	438	278	1.30 (1.21 - 1.37)	0.47 (0.41 - 0.55)	0.349 (0.328 - 0.359)	0.01

Table S8: Number of distances, number of distances used in the fit, parameter values (95% CIs) for the lognormal distributions and p-values for the overall goodness of fit for to the different strata of the commuting distance distributions for Shenzhen employees.

reg stat	sex	age	N	N>1.45	μ	σ	excess <1.45km	p
not reg	m	0-19	136	35	-0.3 (-1991.6 - 1.5)	1.7 (0.9 - 54.9)	0.3 (-∞ - 0.7)	0.23
not reg	m	20-29	1042	535	0.6 (-0.3 - 1.1)	1.5 (1.2 - 1.8)	0.10 (-0.40 - 0.28)	<0.01
not reg	m	30-39	1029	555	1.5 (1.2 - 1.8)	1.31 (1.15 - 1.52)	0.34 (0.24 - 0.39)	<0.01
not reg	m	40-49	528	271	1.3 (0.5 - 1.8)	1.5 (1.2 - 1.9)	0.31 (0.03 - 0.40)	<0.01
not reg	m	50-59	163	67	1.8 (0.8 - 2.3)	1.2 (0.9 - 1.8)	0.54 (0.32 - 0.58)	<0.01
not reg	m	60+	26	8	2.3 (-7.5 - 3.2)	0.9 (0.5 - 4.6)	0.688 (-7.582 - 0.692)	0.62
not reg	f	0-19	154	53	-3 (-1550 - 1)	2.2 (1.0 - 42.3)	-5 (-∞ - 0)	0.86
not reg	f	20-29	835	387	1.12 (0.64 - 1.38)	1.10 (0.94 - 1.36)	0.38 (0.20 - 0.46)	0.17
not reg	f	30-39	637	226	0.8 (-0.5 - 1.3)	1.35 (1.07 - 1.92)	0.44 (-0.13 - 0.56)	<0.01
not reg	f	40-49	333	101	0.3 (-9.1 - 1.2)	1.4 (1.0 - 3.7)	0.37 (-61.38 - 0.62)	0.23
not reg	f	50-59	68	17	-2 (-2665 - 2)	2.1 (0.8 - 75.2)	-0.37 (-∞ - 0.73)	0.25
not reg	f	60+	8	2	(-)	(-)	(-)	
reg	m	0-19	0	0	(-)	(-)	(-)	
reg	m	20-29	136	108	1.7 (1.3 - 2.0)	0.99 (0.80 - 1.34)	0.13 (-0.05 - 0.18)	0.76
reg	m	30-39	248	193	1.7 (1.3 - 2.0)	1.16 (0.96 - 1.47)	0.11 (-0.06 - 0.18)	0.01
reg	m	40-49	169	119	1.7 (0.9 - 2.0)	1.2 (0.9 - 1.6)	0.19 (-0.09 - 0.26)	0.38
reg	m	50-59	58	35	2.2 (1.7 - 2.6)	0.82 (0.61 - 1.26)	0.389 (0.317 - 0.396)	0.83
reg	m	60+	6	1	(-)	(-)	(-)	
reg	f	0-19	1	0	(-)	(-)	(-)	
reg	f	20-29	108	83	1.87 (1.54 - 2.10)	0.82 (0.66 - 1.10)	0.20 (0.12 - 0.23)	0.75
reg	f	30-39	169	123	1.5 (1.1 - 1.8)	0.93 (0.74 - 1.25)	0.19 (-0.01 - 0.24)	0.50
reg	f	40-49	80	53	1.8 (1.4 - 2.1)	0.76 (0.58 - 1.10)	0.318 (0.217 - 0.335)	0.78
reg	f	50-59	16	7	2.3 (1.7 - 2.9)	0.46 (0.25 - 1.15)	0.562 (0.525 - 0.563)	0.97
reg	f	60+	5	3	(-)	(-)	(-)	
mig			1994	209	-446 (-527 - -6)	12.0 (1.5 - 14.3)	-∞ (-∞ - -17468)	<0.01

Analysis of the frequency of occasional travel

If people travelled outside the target area randomly with a given frequency without any difference between individuals, we would expect the number of journeys observed to be distributed across individuals in the population according to a Poisson process with rate $\lambda = \frac{n}{N}$, where n is the number of journeys observed in the population and N is the population size. The expected number N_k of people making k journeys given by

$$N_k = N \frac{\lambda^k e^{-\lambda}}{k!}.$$

Table S9 shows the observed and expected number of people having made 0, 1 or more journeys as well as the p-values of a χ^2-test. The p-values are very small for all study areas, indicating that not all people travel with the same probability, but there are more people not travelling at all, and more people making more than one journey than would be expected.

Table S9: observed and expected number of people having made 0, 1 or more than 1 journeys outside the study area within the previous week for each of the study areas.

	no of people not travelling		no of people making 1 journey		no of people making 2 or more journeys		p-value
	observed	expected	observed	expected	observed	expected	
urban Huangshan	2089	2044	190	253	34	16	$1.7\cdot10^{-8}$
rural Huangshan	7864	7848	221	245	12	3.9	$5.9\cdot10^{-5}$
Shenzhen	11482	11468	376	397	14	7.0	0.016

Gravity model fitting for the occasional travel distance distributions

Reconstruction of the travel distance distributions

For the datasets in Huangshan city, we know the origin of each journey by district (Tunxi district or Xiuning county), for the Shenzhen dataset the origin can be anywhere within Shenzhen city. The destinations of the occasional journeys were recorded at the level of county/district, city or province. Some journeys had several destinations recorded, for the distance distributions these were treated as separate journeys. For each origin-destination pair we calculated a distance distribution by weighting the distances between any two points within the area of origin and the area of destination by both population densities at the points of origin and destination. If there was only one journey for a specific origin-destination pair, this was allocated the median distance, if there were several journeys, they were allocated distances at the relevant percentiles of the distance distribution. The population densities were obtained from the Landscan dataset [2,3] which gives global population density estimates at a resolution of less than 1sqkm.

Binning the distance distributions

The travel distances were binned into bins of logarithmic width, where the maximum distance d_k within distance category k is given by $d_k = f^k d_0$. For a very fine level of aggregation we used $d_0 = 0.5\text{km}$ and $f = 1.05$, yielding around 200 distance categories, many of which had zero

observed journeys. As the chosen value of d_0 was much smaller than the smallest travel distances, we then combined as many categories $k = 0 \ldots k_{\min}$ as was necessary to have at least 5 distances within the first category. We then further aggregated the remaining bins into groups of size n, giving a new aggregation defined by $d_{k'} = \left(f^n \right)^{k'} d_{0'}$ with $d_{0'} = f^{k_{\min}} d_0$. In a last step we then aggregated the last distance categories such that the new last bin contained at least 5 observed journeys.

Dependence of the parameter estimates on the aggregation level

For the results presented in the main paper, we used $n = 12$. Here, we investigate the sensitivity to this arbitrarily chosen level of aggregation. In order to ensure statistical validity of the fitting procedure, we exclude any values of n so small that there are any bins with less than 5 observations. We furthermore exclude values of n so large that there are less than 6 bins.

Figure S4 shows the parameter estimates obtained for different levels of aggregation for the Huangshan and Shenzhen. Although the parameter estimates show a slight variation across the different aggregation levels, they are consistent across the range.

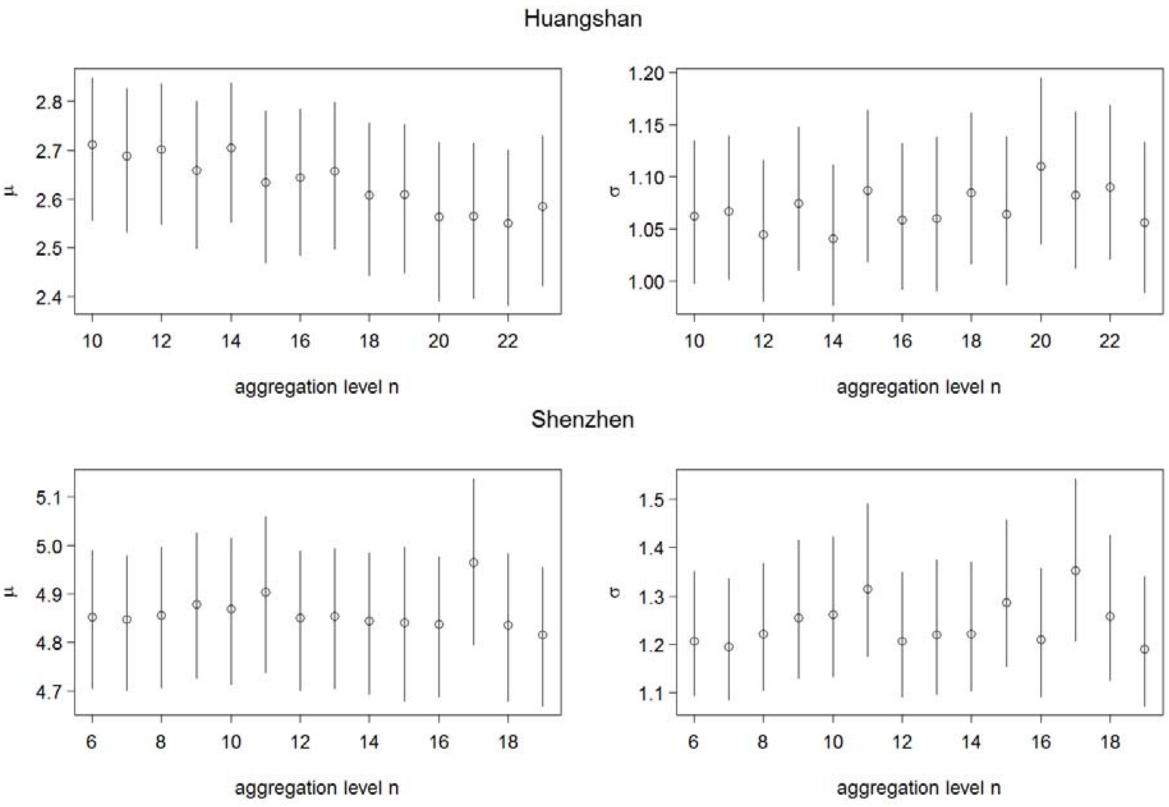

Figure S4: Parameter estimates with 95% confidence intervals for the fitted distance distributions by aggregation level.

Allowing for a destination population power $\tau \neq 1$

When we allow for a variable power on the destination population, the probability of observing a journey within distance interval $[k-1,k]$ is given by

$$p(d_k) = c \cdot k(d) \sum_{i \in \text{origin}} P_i \sum_{j: d_{k-1} < d_{ij} \leq d_k} P_j^{\tau} .$$

With this, we again fitted a lognormal spatial kernel to the observed distance distributions at different levels of spatial aggregation. The obtained parameter estimates are shown in Figure S1.

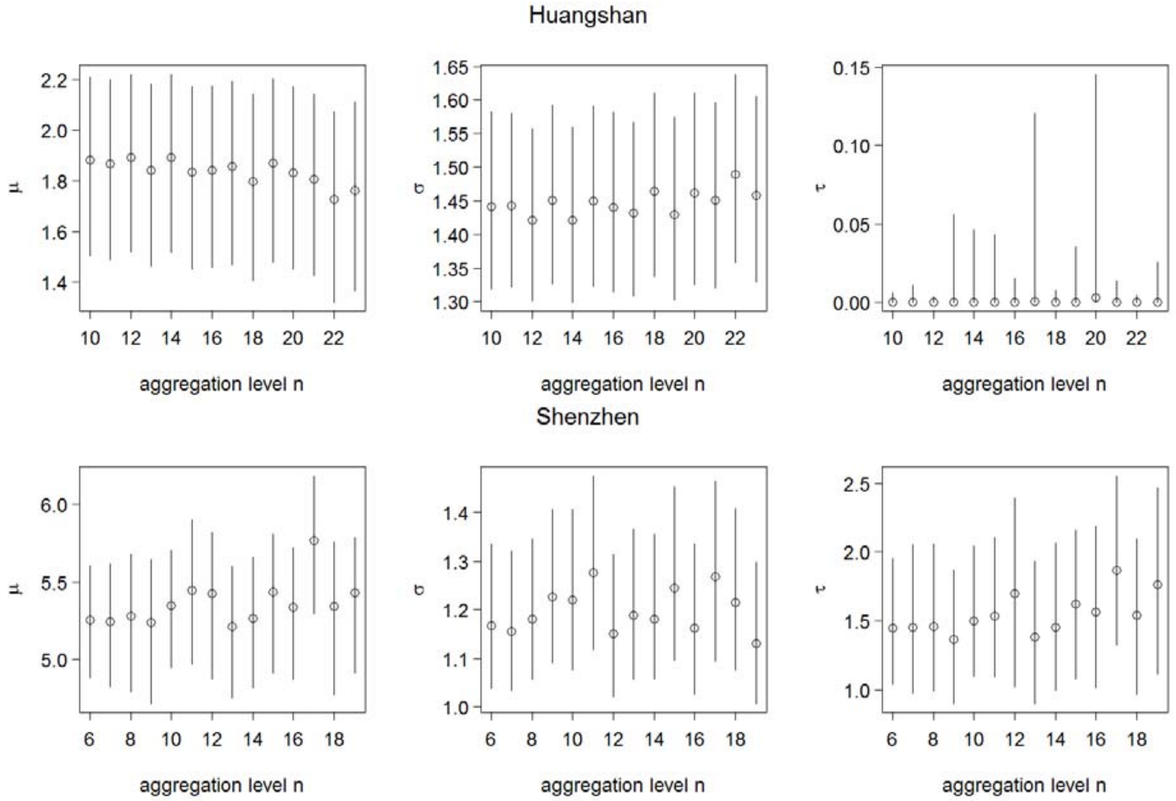

Figure S5: Parameter estimates with 95% confidence intervals for the fitted distance distributions by aggregation level, allowing for a variable power on the destination population density.

For the Shenzhen dataset, the fitted values of the population power τ tend to be somewhat above 1, with 1 being in the area of the lower bound of the confidence interval. This means that the number of people travelling to a particular area is approximately proportional to the population of that area. However, for the Huangshan dataset, the fitted values of the population power τ are very small, indicating that the attractiveness of a travel destination does not depend on its population size. The spatial kernel has a slightly shorter range than with a population power of $\tau = 1$. Comparing the fitted cumulative distributions (Figure S6) with those for the simpler model with the population power fixed to 1 (Figure 7 in the main text), the additional parameter offers only a very slight improvement in the overall fit.

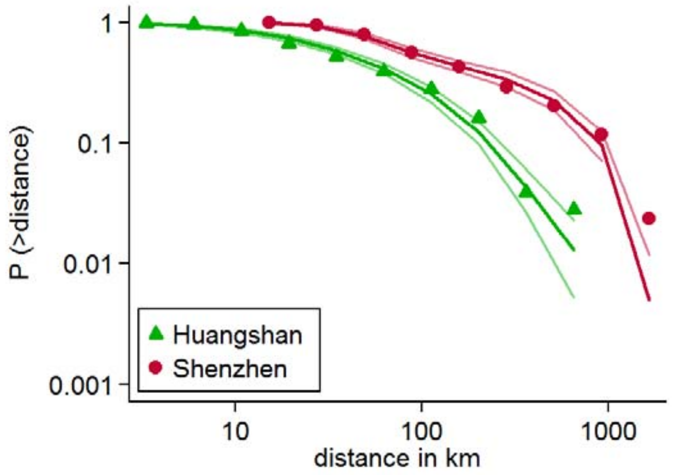

Figure S6: Observed and fitted cumulative distributions of travel distances for Huangshan and Shenzhen. Variable power on the destination population for aggregation level n=12. Symbols = observed distributions, thick lines = fitted distributions, pale lines = 95% credibility intervals of the fitted distributions.

References

1. Hesketh T, Lu L, Xing ZW (2005) The Effect of China's One-Child Family Policy after 25 Years. N Engl J Med 353: 1171-1176.
2. Bhaduri B, Bright E, Coleman P, Dobson J (2002) Landscan: Locating People is What Matters. Geoinformatics 5: 34-37.
3. Dobson JE, Bright EA, Coleman PR, Durfee RC, Worley BA (2000) Landscan: A Global Population Database for Estimating Populations at Risk. Photogrammetric Engineering & Remote Sensing 66: 849-857.
